# Prescribing Pathways Following Private ADHD Diagnosis: An Audit in Peterborough Adult Locality Team

**DOI:** 10.1192/bjo.2026.11661

**Published:** 2026-06-30

**Authors:** Sanaa Moledina, Nirmalan Ayadurai

**Affiliations:** Cambridgeshire and Peterborough NHS Foundation Trust, Cambridge, United Kingdom

## Abstract

**Aims:**

The demand for adult Attention Deficit Hyperactivity Disorder (ADHD) assessments has surged while NHS capacity remains constrained by limited budgets, workforce shortages, and uneven specialist provision. Lack of national standardisation in ADHD pathways has created regional disparities, with waiting times ranging from two to five years–often termed the “ADHD postcode lottery.” Many patients turn to private clinics offering faster assessments, either self-funded or via the NHS Right to Choose pathway, which allows eligible patients to access approved private providers at NHS expense.

NICE guidelines (2018) state that “after titration and dose stabilisation, prescribing and monitoring of ADHD medication should be carried out under shared care protocol arrangements with primary care.” However, shared care implementation is inconsistent, with reported concerns among clinicians about private assessment validity and medicolegal implications of prescribing medications initiated outside NHS services. Without shared care arrangements, patients must either continue costly private prescribing or re-join NHS waiting lists for reassessment and treatment, risking abrupt treatment discontinuation.

This audit aimed to identify the prevalence of private ADHD diagnoses within the North Peterborough Adult Locality Team (PALT) caseload, determine how prescribing responsibility is managed, and assess alignment with NICE shared care guidance to inform local policy development.

**Methods:**

Patient records were reviewed retrospectively using clinic letters and keyword searches (e.g., “ADHD,” “private,” “Right to Choose,” “shared care”). Data collected included demographics, route of diagnosis, treatment status, prescribing arrangements, and shared care documentation.

**Results:**

Of the 325 patients reviewed, 2 (0.6%) had received private ADHD diagnoses, both in 2022. Both were female, aged 39 and 47, one British and one African-Asian. The first accessed diagnosis via NHS Right to Choose and was managed under shared care between her private psychiatrist and GP for two years until discontinuing medication. The second obtained a self-funded private diagnosis but reverted to the NHS ADHD clinic treatment waitlist due to costs; subsequent delays in accessing treatment led to her returning to private care.

**Conclusion:**

We identified a low prevalence of private ADHD diagnoses within the caseload. While the smaller cohort precludes generalisation, Right to Choose facilitated successful shared care, whereas self-funded pathways resulted in treatment delays. No mechanism exists for privately diagnosed patients to access NHS treatment without joining standard waiting lists, undermining the rationale for expedited private diagnosis and perpetuating treatment gaps. Further audit in a larger cohort is recommended to inform local policy development.

